# Plasmids Shape the Current Prevalence of *tmexCD1-toprJ1* among Klebsiella pneumoniae in Food Production Chains

**DOI:** 10.1128/mSystems.00702-21

**Published:** 2021-10-05

**Authors:** Kai Peng, Qian Wang, Yi Yin, Yan Li, Yuan Liu, Mianzhi Wang, ShangShang Qin, Zhiqiang Wang, Ruichao Li

**Affiliations:** a College of Veterinary Medicine, Yangzhou University, Yangzhou, Jiangsu Province, People’s Republic of China; b Jiangsu Co-innovation Center for Prevention and Control of Important Animal Infectious Diseases and Zoonoses, Yangzhou, Jiangsu Province, People’s Republic of China; c Institute of Comparative Medicine, Yangzhou University, Yangzhou, Jiangsu Province, People’s Republic of China; d School of Pharmaceutical Sciences, Zhengzhou University, Zhengzhou, Henan Province, People’s Republic of China; e Key Laboratory of Advanced Drug Preparation Technologies, Ministry of Education, Zhengzhou University, Zhengzhou, Henan Province, People’s Republic of China; California State University, Fresno

**Keywords:** tigecycline resistance, *tmexCD1-toprJ1*, tandem repeats, plasmids

## Abstract

The emergence of novel antimicrobial resistance genes conferring resistance to last-resort antimicrobials poses a serious challenge to global public health security. Recently, one plasmid-mediated RND family multidrug resistance efflux pump gene cluster named *tmexCD1-toprJ1*, which confers resistance to tigecycline, was identified in bacteria of animal and human origins. However, the comprehensive landscape of the genomic epidemiology of this novel resistance determinant remained unclear. To fill this knowledge gap, we isolated 25 *tmexCD1-toprJ1*-positive bacteria from 682 samples collected along the pork production chain, including swine farms, slaughterhouses, and retail pork, and characterized the positive strains systematically using antimicrobial susceptibility testing, conjugation assays, single-molecule sequencing, and genomic analyses. We found that *tmexCD1-toprJ1*-positive bacteria were most prevalent in slaughterhouses (7.32%), followed by retail pork (0.72%). Most of the positive strains were Klebsiella pneumoniae (23/25), followed by Proteus mirabilis (2/25). IncFIB(Mar)/IncHI1B hybrid plasmids were mainly vectors for *tmexCD1-toprJ1* and dominated the horizontal dissemination of *tmexCD1-toprJ1* among K. pneumoniae isolates. However, in this study, we identified the IncR plasmid as a *tmexCD1-toprJ1*-positive plasmid with a broad host range, which evidenced that the widespread prevalence of *tmexCD1-toprJ1* is possible due to such kinds of plasmids in the future. In addition, we found diversity and heterogeneity of translocatable units containing *tmexCD1-toprJ1* in the plasmids. We also investigated the genetic features of *tmexCD1-toprJ1* in online databases, which led to the proposal of the *umuC* gene as the potential insertion site of *tmexCD1-toprJ1*. Collectively, this study enriches the epidemiological and genomic characterization of *tmexCD1-toprJ1* and provides a theoretical basis for preventing an increase in *tmexCD1-toprJ1* prevalence.

**IMPORTANCE** Tigecycline, the first member of the glycylcycline class of antibacterial agents, is frequently used to treat complicated infections caused by multidrug-resistant Gram-positive and Gram-negative bacteria. The emergence of a novel plasmid-mediated efflux pump, TmexCD1-ToprJ1, conferring resistance to multiple antimicrobials, including tigecycline, poses a huge risk to human health. In this study, we investigated the prevalence of *tmexCD1-toprJ1*-positive strains along the food production chain and found that *tmexCD1-toprJ1* was mainly distributed in IncFIB(Mar)/HI1B hybrid plasmids of K. pneumoniae. We also observed a potential risk of transmission of such plasmids along the pork processing chain, which finally may incur a threat to humans. Furthermore, the IncFIB(Mar)/HI1B *tmexCD1-toprJ1*-positive plasmids with a limited host range and specific insertion sites of *tmexCD1-toprJ1* are strong evidence to prevent a fulminant epidemic of *tmexCD1-toprJ1* among diverse pathogens. The mobilization and dissemination of *tmexCD1-toprJ1*, especially when driven by plasmids, deserve sustained attention and investigations.

## INTRODUCTION

Antimicrobial resistance is a global concern and recognized as one of the greatest threats to public health worldwide (1). The misuse, abuse, and widespread use of antimicrobials in clinical and livestock settings account for the emergence and explosion of antimicrobial resistance. There is an urgent need for the development of effective interventions to control the hazard caused by the dissemination of resistance genes (2). Although nonsusceptible bacteria can evade the bactericidal effect driven by their intrinsic resistance, reports of acquired resistance via horizontal gene transfer are indicative of antimicrobial resistance genes moving flexibly within and between species, which becomes the predominant reason behind the emergence of multidrug-resistant (MDR) bacteria (3, 4). Many mobile elements with horizontal transfer capacity, including plasmids, integrative conjugative elements (ICEs), and transposons, were associated with antibiotic resistance genes (ARGs) (3, 5, 6). Plasmids, extrachromosomal DNA elements with the ability to self-replicate, have emerged as the most crucial mobile element since they are the most common vehicle for capturing and mobilizing ARGs (7). The universal prevalence of many clinically critical resistance genes, such as *bla*_NDM_ (8, 9), *mcr* (10, 11), and *tet*(X) (12), was associated with the horizontal transmission of various plasmids. Uncovering the characteristics of different plasmids and mobile elements can reveal novel transfer pathways of ARGs, which would benefit their control.

Tigecycline, a semisynthetic glycylcycline derivative of tetracycline (13), is one of the last-resort antimicrobials to treat serious infections caused by MDR bacteria (14). The overexpression of chromosomally encoded nonspecific efflux pumps or mutations within the drug-binding site in the ribosome play a pivotal role in tigecycline resistance (15–18). In addition, the occurrence of plasmid-mediated *tet*(X) family genes encoding tetracycline-inactivated enzymes further diminishes the efficacy of tigecycline (19, 20). The diversity and polymorphism of *tet*(X)-harboring plasmids were identified from disparate species of bacteria shortly after the mechanism of plasmid-mediated resistance gene transfer was first reported (12, 21, 22). Moreover, a plasmid-borne RND family multidrug efflux pump gene cluster called *tmexCD1-toprJ1* was recently discovered in Klebsiella pneumoniae of animal origin, showing no susceptibility to tigecycline (23). This implies that two different mechanisms of plasmid-mediated tigecycline resistance that occurred in succession have undermined the efficacy and use of tigecycline.

Unlike *tet*(X) genes, plasmid-borne *tmexCD1-toprJ1* has rarely been reported since its discovery. Even though the only recently described host of *tmexCD1-toprJ1* was K. pneumoniae, the corresponding gene cluster was not infrequent in the NCBI database, even though its host range remains unexplored. Previous research considered that the gene cluster originated from Pseudomonas spp., according to genomic analysis of data in the NCBI database (23, 24). To date, no research has been found that explores the large-scale prevalence of *tmexCD1-toprJ1* in *Enterobacterales* of animal sources, and detailed genetic features of *tmexCD1-toprJ1* have scarcely been investigated. Therefore, we aimed to investigate the prevalence of *tmexCD1-toprJ1* in *Enterobacterales* in the pork production chain, from swine farms to retail pork. Simultaneously, data released in the NCBI nr database associated with *tmexCD1-toprJ1* were downloaded and analyzed. This enabled the assessment of characteristics of *tmexCD1-toprJ1-*bearing plasmids from *Enterobacterales* and the core genetic environment of *tmexCD1-toprJ1* from different bacteria.

## RESULTS AND DISCUSSION

### Prevalent features of *tmexCD1-toprJ1*-bearing *Enterobacterales* in the pork production chain.

During a survey evaluating the prevalence of *tmexCD1-toprJ1* in *Enterobacterales* from the swine industry, a total of 25 *tmexCD1-toprJ1*-positive strains, consisting of 23 K. pneumoniae and 2 Proteus mirabilis strains, were isolated from 682 samples, including retail pork, feces, soil, carcass, sewage, blood, and dust. These samples were collected from different wet markets, slaughterhouses, and farms in various regions of China (see Table S1 in the supplemental material). The comprehensive positivity rate was 3.37%, which was lower than the previously reported prevalences of *tet*(X) of animal origin (28.33% and 6%) (12, 22). Of the 25 positive strains, 24 were isolated from swine slaughterhouses, and 1 was isolated from retail pork. No positive strains were detected in samples from swine farms. The most prevalent occurrence of *tmexCD1-toprJ1* was found in a slaughterhouse in Nantong city, Jiangsu, followed by another slaughterhouse in Yangzhou city, Jiangsu. In contrast, only one positive strain was isolated from retail pork, and none were recovered from swine farms (Fig. S1 and Table S1). Notably, a higher prevalence of *tet*(X) genes was observed in the Nantong slaughterhouse than in Nantong swine farms from our previous studies (12, 22). These findings indicated that the prevalence of resistance genes with the same resistance phenotype was positively associated with their environmental background. Although tigecycline was not licensed for use in animals, tetracyclines have been used in the livestock industry for many years in China. The long-term use of tetracyclines in livestock might be the cause of the emergence and prevalence of *tmexCD1-toprJ1*. Slaughterhouses are important links in the animal food processing chain as well as a transfer station for the convergence of livestock from different regions, thus becoming a potential reservoir for various pathogenic MDR bacteria (25, 26). The high detection rate of *tmexCD1-toprJ1* in slaughterhouses seems to be associated with horizontal gene transfer between bacteria from different niches. Furthermore, a strain positive for *tmexCD1-toprJ1* was detected in retail pork, suggesting that animal-derived bacteria could spread to humans through the food production chain.

10.1128/mSystems.00702-21.1 FIG S1 Detection rates of *tmexCD1-toprJ1*-positive strains in different source samples. Download FIG S1, TIF file, 0.2 MB. Copyright © 2021 Peng et al. 2021 Peng et al. https://creativecommons.org/licenses/by/4.0/ This content is distributed under the terms of the Creative Commons Attribution 4.0 International license.

10.1128/mSystems.00702-21.5 TABLE S1 Samples collected from different sources and prevalence of *tmexCD1-toprJ1* in these samples. Download Table S1, DOCX file, 0.02 MB. Copyright © 2021 Peng et al. 2021 Peng et al. https://creativecommons.org/licenses/by/4.0/ This content is distributed under the terms of the Creative Commons Attribution 4.0 International license.

Species identification showed that most of the positive strains (23/25) were K. pneumoniae, followed by Proteus mirabilis (2/25). This implies that the prevalence of *tmexCD1-toprJ1* might currently be limited by host adaptation and that the potential risk of the further diffusion of *tmexCD1-toprJ1* by diverse bacterial species was limited. Our results revealed that all 25 *tmexCD1-toprJ1*-positive strains were resistant to tigecycline, with MICs from 16 to 32 mg/liter. Furthermore, the addition of the efflux pump inhibitor 1-(1-naphthylmethyl)-piperazine (NMP) significantly reduced the tigecycline MIC against these strains by 8-fold (Table S2). Therefore, the resistance to tigecycline in these strains was mediated by *tmexCD1-toprJ1*. In addition to tigecycline, all K. pneumoniae strains were resistant to minocycline, oxytetracycline, doxycycline, tetracycline, amoxicillin, kanamycin, and streptomycin but susceptible to meropenem and colistin (Table S2).

10.1128/mSystems.00702-21.6 TABLE S2 Antimicrobial susceptibility testing (MICs [milligrams per liter]) of 23 K. pneumoniae isolates, 2 P. mirabilis isolates, and 1 transconjugant harboring the *tmexCD1-toprJ1* gene cluster. Download Table S2, DOCX file, 0.03 MB. Copyright © 2021 Peng et al. 2021 Peng et al. https://creativecommons.org/licenses/by/4.0/ This content is distributed under the terms of the Creative Commons Attribution 4.0 International license.

### Genomic epidemiology of *tmexCD1-toprJ1*-positive isolates.

**FIG 1:**
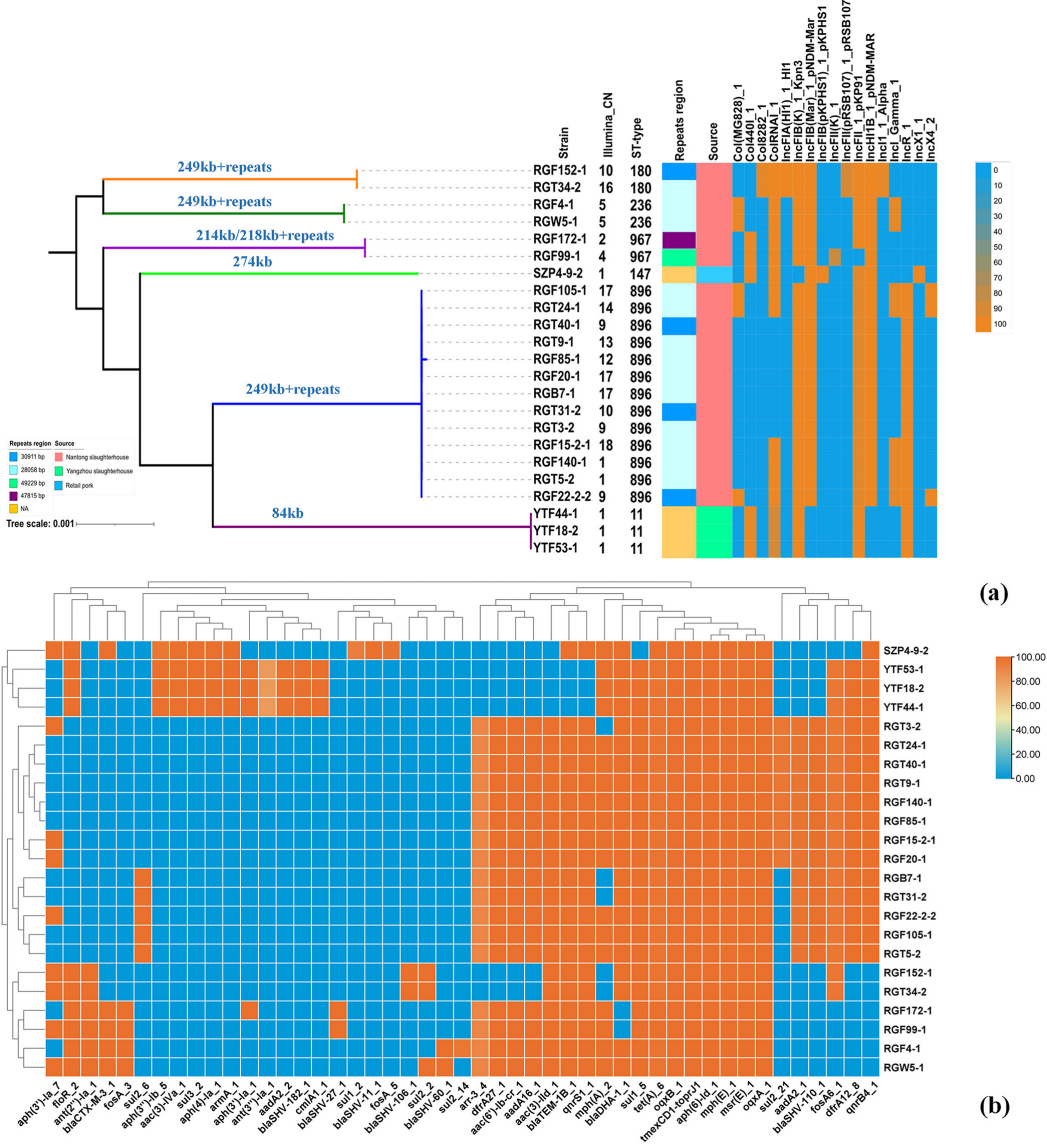
Phylogenetic analysis and basic information for the 23 isolated *tmexCD1-toprJ1*-positive K. pneumoniae strains. (a) A phylogenetic tree was constructed using Roary and FastTree based on core-genome SNPs. Captions in blue on the branches show the size of *tmexCD1-toprJ1*-bearing plasmids in the strains of that branch. CN represents the copy number of *tmexCD1-toprJ1* in the genome of the corresponding strain. NA, not applicable. (b) Distribution of resistance genes in the 23 *tmexCD1-toprJ1*-positive K. pneumoniae strains.

To investigate the genetic characterization of the 25 *tmexCD1-toprJ1*-positive strains, we performed whole-genome sequencing (WGS) using the Illumina HiSeq platform. The draft genome was obtained by a *de novo* assembly strategy with SPAdes v0.4.8. We then constructed a phylogenetic tree consisting of 23 K. pneumoniae isolates based on single nucleotide polymorphisms (SNPs) of core genomes (Fig. 1a). The 23 K. pneumoniae isolates were clustered into 6 clonal groups, and the diversity in every cluster was imperceptible. In every cluster, fewer than 300 SNPs between strains were found (Fig. S2), thus suggesting phylogenetically high similarity within each cluster. Multilocus sequence typing (MLST) analysis showed that the six clusters corresponded to six sequence types (STs) (ST180, ST236, ST967, ST147, ST896, and ST11). ST896 was the predominant ST (*n* = 13), followed by ST11 (*n* = 3), ST180 (*n* = 2), ST236 (*n* = 2), ST967 (*n* = 2), and ST147 (*n* = 1). Significantly, the 13 strains that belonged to ST896 were isolated from different samples, which included feces, carcasses, and blood. This revealed that there was a possibility of cross-contamination between samples, resulting in a regional epidemic of *tmexCD1-toprJ1*-positive ST896 K. pneumoniae within the slaughterhouse. Previous studies mentioned that carbapenem-resistant ST896 K. pneumoniae had been involved in nosocomial infections (27, 28). Hence, effective measures should be advocated to prevent the dissemination of *tmexCD1-toprJ1*-positive ST896 K. pneumoniae. Furthermore, K. pneumoniae strains of ST147 and ST11 were often associated with different carbapenemases and virulence genes, making them a high-risk clinical clone globally (29, 30). As a result, the emergence of *tmexCD1-toprJ1*-positive ST147 and ST11 K. pneumoniae strains poses a great threat to human public health.

10.1128/mSystems.00702-21.2 FIG S2 Numbers of SNPs in the 23 Klebsiella pneumoniae strains. The SNPs were analyzed using snp-dists based on core genes of the 23 Klebsiella pneumoniae strains. The similar curves indicate that the strains originated from one clone. The numerical values indicate SNPs between different strains. Download FIG S2, TIF file, 0.9 MB. Copyright © 2021 Peng et al. 2021 Peng et al. https://creativecommons.org/licenses/by/4.0/ This content is distributed under the terms of the Creative Commons Attribution 4.0 International license.

Subsequently, we analyzed the distribution of plasmids and resistance genes in the 23 K. pneumoniae strains. The strains with identical STs presented similar plasmid profiles and resistance genes (Fig. 1a). In addition to *tmexCD1-toprJ1*-bearing plasmids, other plasmids with various resistance genes were found in these strains. Notably, an extended-spectrum beta-lactamase (ESBL) gene named *bla*_CTX-M-3_ was detected in five strains (Fig. 1b). The abundant resistance genes and the diversity of plasmids in these strains reflected that *tmexCD1-toprJ1*-positive MDR K. pneumoniae strains have strong adaptability to a hostile environment, especially due to the wide use of tetracyclines in livestock.

Apart from K. pneumoniae, two P. mirabilis strains were isolated from different fecal samples collected in the Nantong slaughterhouse. They harbored a *tmexCD1-toprJ1-*like gene cluster, designated *tmexCD3-toprJ3*. One of the strains, named RGF134-1, has been investigated in detail in our previous work (31). The *tmexCD3-toprJ3* gene cluster was located in an SXT/R391 family integrative conjugative element (ICE). The emergence of *tmexCD3-toprJ3*-positive P. mirabilis demonstrated that cross-species transmission of the *tmexCD1-toprJ1* family gene cluster and *in situ* evolution might have occurred in a slaughterhouse. Moreover, ICEs are also important carriers for the horizontal transfer of resistance genes (32). The transfer of ICEs between diverse bacteria, particularly to or from Proteus spp., further broadened the host range of the *tmexCD1-toprJ1* family gene cluster. Further investigation of the horizontal transfer of the *tmexCD1-toprJ1* family gene cluster is needed to better understand the transmission mechanisms.

### Transmissibility of *tmexCD1-toprJ1*-bearing genetic structures.

In order to test the transmissibility of the *tmexCD1-toprJ1* gene cluster, all positive strains were subjected to a conjugation assay with Escherichia coli J53 and E. coli C600 as recipients. However, no positive transconjugants were recovered after three attempts. Subsequently, we selected representative strains susceptible to hygromycin to perform a conjugation assay with hygromycin-resistant ST11 K. pneumoniae HS11286YZ6 as the recipient strain (33). The *tmexCD1-toprJ1*-bearing plasmids in strains RGT40-1 and RGF105-1 were successfully transferred into HS11286YZ6, and the transconjugants showed a 16- to 32-fold increase of the MICs of tigecycline (Table S2). This result demonstrated that these *tmexCD1-toprJ1*-positive plasmids can be transferred conjugatively, although this is limited by the host range. This is consistent with our observations from a previous study (33). In addition, we selected three *tmexCD1-toprJ1*-positive plasmids of different sizes, namely, pRT40-1-tmexCD, pRGF99-1-tmexCD, and pTF44-1-tmexCD, to perform electrotransformation with E. coli DH5α as the recipient. The aim of this experiment was to further verify the transferability of *tmexCD1-toprJ1*-positive plasmids. The three plasmids were successfully transferred into DH5α, which resulted in DH5α with *tmexCD1-toprJ1*-positive plasmids exhibiting resistance to tigecycline with MICs of 8 mg/liter. What is noteworthy is that E. coli harboring *tmexCD1-toprJ1* showed lower-level resistance to tigecycline than *tmexCD1-toprJ1*-positive K. pneumoniae (Table S2). Additionally, a previous study demonstrated that the expression of TMexCD1-TOprJ1 had negative effects on E. coli growth (23). Therefore, it is reasonable that the *tmexCD1-toprJ1* gene cluster tended to be more prevalent in K. pneumoniae.

### Distribution of *tmexCD1-toprJ1-*bearing plasmids in Klebsiella spp.

**FIG 2:**
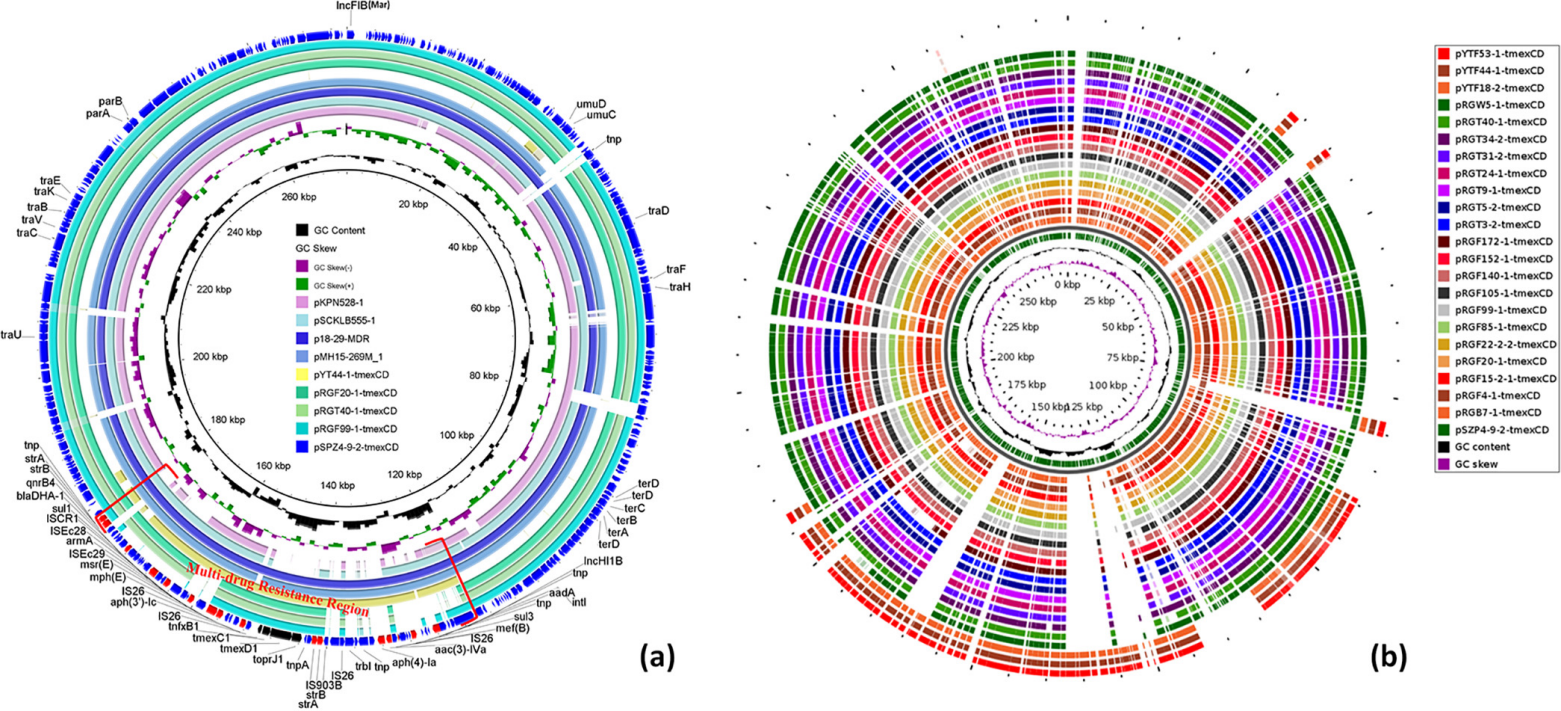
Comparison analysis of *tmexCD1-toprJ1*-bearing plasmids. (a) Comparison analysis between *tmexCD1-toprJ1*-bearing plasmids and other similar plasmids. (b) Structure analysis of *tmexCD1-toprJ1*-positive plasmids in this study. The structural diversity of these plasmids existed within an MDR region.

In order to explore the genetic diversity of *tmexCD1-toprJ1* in K. pneumoniae, we selected 14 different strains from different clusters in the phylogenetic tree to obtain the complete genome using the Nanopore MinION sequencing platform. Most of the *tmexCD1-toprJ1*-bearing plasmids were assembled into two contigs, although these were not circular, resulting from tandem repeats of the *tmexCD1-toprJ1*-containing region. We obtained only two complete *tmexCD1-toprJ1-*bearing plasmids, named pYTF44-tmexCD and pSZP4-9-2-tmexCD (sizes of 84 kb and 274 kb, respectively), which were carried by YTF44-1 and SZP4-9-2, directly by a hybrid assembly strategy. The 84-kb plasmid pYTF44-tmexCD showed 72% coverage and 99.98% homology with pSZP4-9-2-tmexCD (Fig. 2a). Interestingly, analysis of the plasmid replicon type indicated that pYTF44-tmexCD was classified as the IncR type, while pSZP4-9-2-tmexCD harbored two replicon genes belonging to the IncFIB(Mar)/IncHI1B hybrid type. The coverage region of the two plasmids was a multidrug resistance region (MRR) containing *tmexCD1-toprJ1* (Fig. 2a). Thus, we presumed that the MRR was mobile and had the capacity to integrate into different plasmids.

Further analysis revealed that all *tmexCD1-toprJ1*-positive plasmids that we detected could be categorized as the two types of plasmids mentioned above. The 84-kb pYTF44-tmexCD-like plasmids were detected in only 3 strains of ST11 from the Yangzhou slaughterhouse, whereas the 274-kb pSZP4-9-2-tmexCD-like plasmids were widely distributed in 20 strains of different STs (Fig. 2b). Notably, SZP4-9-2 was isolated from retail pork, while other strains harboring pSZP4-9-2-tmexCD-like plasmids were isolated from the slaughterhouse in Nantong, indicating that such plasmids can potentially be transmitted along the food production chain. Furthermore, pSZP4-9-2-tmexCD-like plasmids were detected in 13 strains of ST896, which suggested that the spread of *tmexCD1-toprJ1* by bacterial division is also worth taking into account. All *tmexCD1-toprJ1-*bearing plasmids that failed to assemble were detected in strains isolated from the Nantong slaughterhouse, and they shared similar backbones with pSZP4-9-2-tmexCD. However, the repeat region containing *tmexCD1-toprJ1* was diverse in these plasmids. Three representative plasmids (pRGF99-1-tmexCD, pRGT40-1-tmexCD, and pRGF20-1-tmexCD) containing three types of *tmexCD1-toprJ1* repeat units with different copies were assembled manually, and the number of repeat units was kept at 2. We observed significant differences among these pSZP4-9-2-tmexCD-like plasmids solely in terms of the resulting MRR (Fig. 2a). This phenomenon illustrated that the MRR was variable and that the repeat region could change, leading to greater diversity.

**FIG 3:**
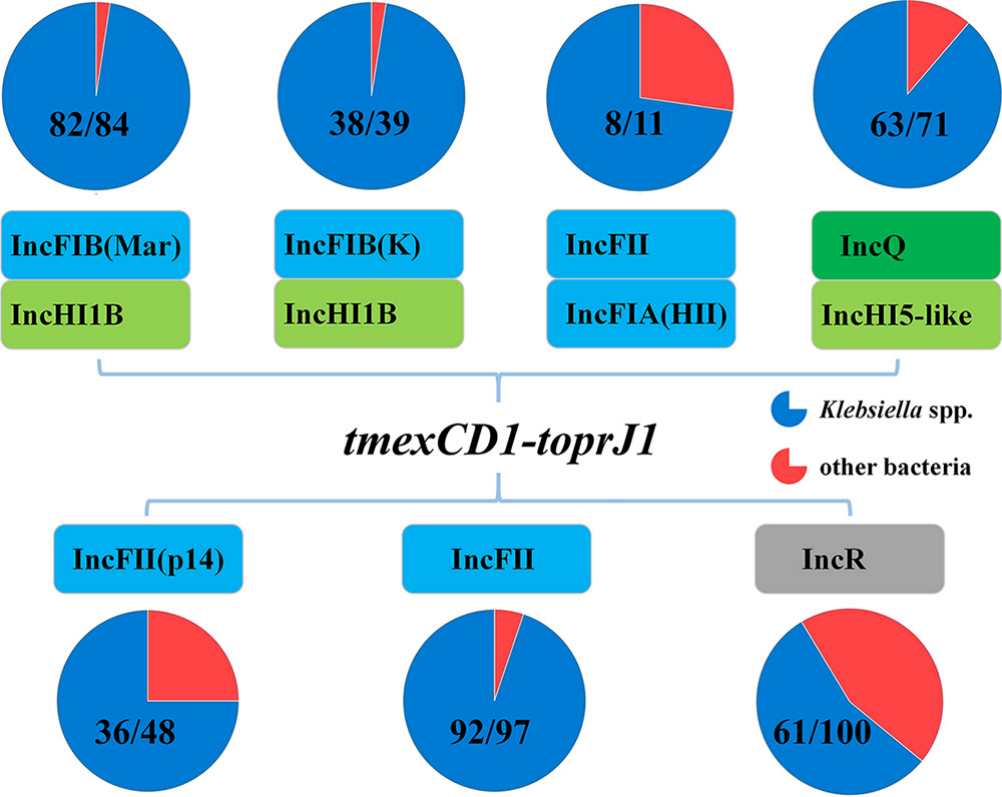
Replicon types of *tmexCD1-toprJ1*-positive plasmids in K. pneumoniae and host range analysis of these plasmids. A total of seven types of plasmids harboring *tmexCD1-toprJ1* have been detected in K. pneumoniae to date. Colored boxes represent different plasmid replicons. Stacked boxes indicate hybrid plasmids that harbor more than one replicon. The host ranges of the plasmids were determined by BLASTn analysis against plasmids with the same replicon in the NCBI nr database and are displayed in pie diagrams. The fractions in the pie diagrams indicate the numbers of corresponding types of plasmids harbored by *Klebsiella* spp. and other bacteria in the NCBI nr database.

Subsequently, we analyzed the host range of these two types of plasmids using BLASTn analysis against plasmids with the same replicon in the NCBI nr database. The IncFIB(Mar)/IncHI1B-type plasmids were carried mostly by Klebsiella spp., whereas the IncR-type plasmids were widely distributed in a variety of bacteria (Fig. 3). Therefore, these IncFIB(Mar)/IncHI1B-type plasmids tended to be prevalent in Klebsiella spp. The *tmexCD1-toprJ1*-containing region was lacking in most IncFIB(Mar)/IncHI1B-type plasmids from the NCBI nr database (Fig. 2a), indicating that the *tmexCD1-toprJ1* gene cluster was integrated into this type of plasmid to aid host bacteria against antimicrobial pressure. Additionally, we found in the NCBI nr database that two *tmexCD1-toprJ1*-positive plasmids, p18-29-MDR and pMH15-296_M, shared a similar backbone with pSZP4-9-2-tmexCD (Fig. 2a). Notably, the two plasmids were isolated from patients, and pMH15-296_M was first discovered in 2015 (Table S3). This phenomenon indicated that such plasmids have led to the dissemination of *tmexCD1-toprJ1* for many years. Hence, IncFIB(Mar)/IncHI1B-type plasmids seemed to be primary receptacles for *tmexCD1-toprJ1* and dominated the horizontal dissemination of *tmexCD1-toprJ1* among pathogens, especially among Klebsiella spp. Despite the low prevalence of IncR-type *tmexCD1-toprJ1*-positive plasmids, their emergence increased the risk of *tmexCD1-toprJ1* spreading to other genera of bacteria.

10.1128/mSystems.00702-21.7 TABLE S3 Basic information for *tmexCD1-toprJ1*-bearing sequences in the NCBI nr database. Download Table S3, DOCX file, 0.03 MB. Copyright © 2021 Peng et al. 2021 Peng et al. https://creativecommons.org/licenses/by/4.0/ This content is distributed under the terms of the Creative Commons Attribution 4.0 International license.

To characterize all available *tmexCD1-toprJ1*-bearing plasmids in Klebsiella spp., 12 *tmexCD1-toprJ1*-positive plasmids were downloaded from the NCBI nr database (as of 20 January 2021) (Table S3). These plasmids, with sizes ranging from 102,569 bp to 375,474 bp, were categorized into five groups according to plasmid backbone similarity (Fig. 2a and Fig. S3). These types of plasmids were carried by Klebsiella spp. in almost every case, and most of them harbored more than one replicon gene (Fig. 3). The variety of *tmexCD1-toprJ1*-bearing plasmids found in Klebsiella spp. implied that the *tmexCD1-toprJ1* gene cluster was prevalent in Klebsiella spp. due to these plasmids. In addition, the limited host range of these plasmids is consistent with the fact that *tmexCD1-toprJ1* was rarely found in other genera of *Enterobacterales*. However, the potential risk of *tmexCD1-toprJ1* being transferred to other species of bacteria by plasmids with a broad host range, such as IncR-type plasmids, deserves continued monitoring. Furthermore, most *tmexCD1-toprJ1*-positive Klebsiella species strains (9/12) were also identified in human patients (Table S3), which might be related to the frequent use of tigecycline in the clinic. The appropriate use of antibiotics is critical to slow the emergence of antimicrobial resistance.

10.1128/mSystems.00702-21.3 FIG S3 Circular comparison of *tmexCD1-toprJ1*-bearing plasmids with their similar plasmids. (a) Comparative analysis of four *tmexCD1-toprJ1*-positive plasmids with a size of ∼120 kb with a *tmexCD1-toprJ1*-negative plasmid, pKP91 (GenBank accession no. MG736312). (b) Comparative analysis of three *tmexCD1-toprJ1*-positive plasmids with a size of ∼270 kb with a *tmexCD1-toprJ1*-negative plasmid, p10057-catA (GenBank accession no. MN423364). (c) Circular comparison of pKP19-3088-375kb with its variant pKP19-3023-374kb and a *tmexCD1-toprJ1*-positive plasmid, pNDM-1-Ec12 (GenBank accession no. MN598004). (d) Circular comparison of pKP19-3088-159kb with its variant pKP19-3023-142kb and a *tmexCD1-toprJ1*-positive plasmid, pZZ40-KPC (GenBank accession no. MN891679). The horizontal transfer of *tmexCD1-toprJ1* occurred in pKP19-3088-375kb and pKP19-3088-159kb. Download FIG S3, TIF file, 1.7 MB. Copyright © 2021 Peng et al. 2021 Peng et al. https://creativecommons.org/licenses/by/4.0/ This content is distributed under the terms of the Creative Commons Attribution 4.0 International license.

### Core genetic structures of *tmexCD1-toprJ1* in plasmids of Klebsiella spp.

**FIG 4:**
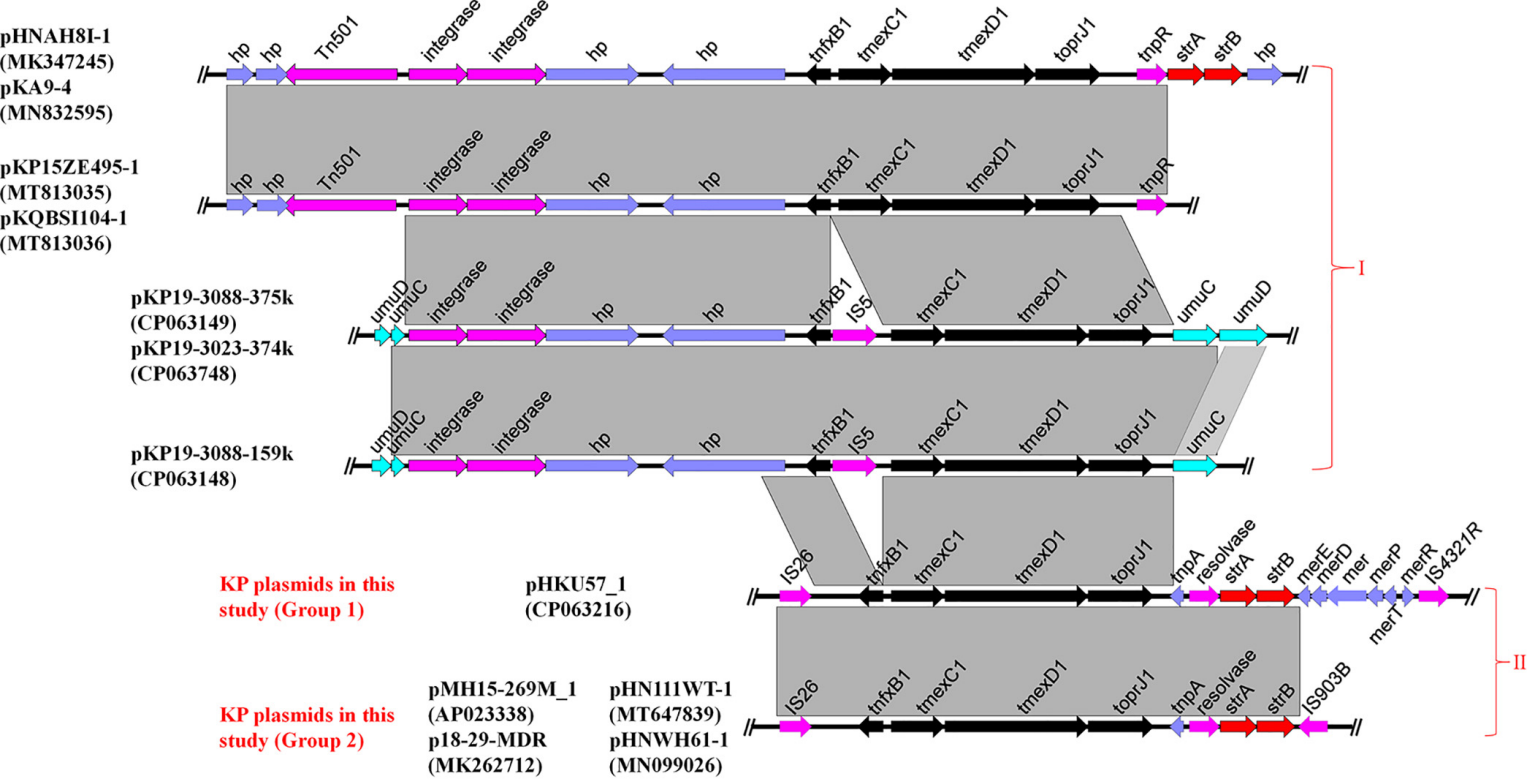
Core genetic structures of *tmexCD1-toprJ1* in plasmids carried by Klebsiella spp. Group 1 consists of plasmids found in strains isolated from a slaughterhouse in Nantong. Group 2 consists of plasmids carried by strains isolated from retail pork and a slaughterhouse in Yangzhou. KP, K. pneumoniae.

To investigate the core genetic environment of *tmexCD1-toprJ1* in all positive Klebsiella species strains, *tmexCD1-toprJ1*-containing regions from all *tmexCD1-toprJ1*-bearing plasmids in Klebsiella spp. were analyzed. The core genetic structures of *tmexCD1-toprJ1* in Klebsiella spp. were clustered into two types (Fig. 4). The distinction of type I and type II was the presence of two integrase genes upstream of *tmexCD1-toprJ1* (Fig. 4). These two integrases were considered to be involved in the transmission of the *tmexCD1-toprJ1* gene cluster (23).

The type I genetic context (with gene arrangements *int-int-hp-hp-tnfxB1*-*tmexCD1-toprJ1* and *int-int-hp-hp-tnfxB1-*IS*5*-*tmexCD1-toprJ1*) was observed in most *tmexCD1-toprJ1*-bearing plasmids of Klebsiella spp. Different insertion sites of the genetic context were found in different plasmids (Fig. 4), which further confirmed that the two integrases can cotransfer the *tmexCD1-toprJ1* gene cluster. An IS*5* element was inserted into *tnfxB1*-*tmexCD1-toprJ1* in plasmids pKP19-3088-375k, pKP19-3023-374k, and pKP19-3088-159k; however, it did not affect the mobilization of *int-int-hp-hp-tnfxB1-*IS*5*-*tmexCD1-toprJ1* (34).

The core genetic environment of type II featured an IS*26* element located upstream of *tmexCD1-toprJ1*, which has the ability to capture and mobilize *tmexCD1-toprJ1* (35). The core genetic environments of *tmexCD1-toprJ1* in all detected *tmexCD1-toprJ1* plasmids were associated with IS*26* (Fig. 4). The discovery of IS*26*-mediated *tmexCD1-toprJ1* demonstrated that diverse plasmids or mobile elements can acquire *tmexCD1-toprJ1* by IS*26* and thus exacerbate the spread of *tmexCD1-toprJ1* in different bacteria. The IS*26*-mediated core genetic context of *tmexCD1-toprJ1* can be further categorized into two subtypes, according to the following genetic arrangements: IS*26-tnfxB1*-*tmexCD1-toprJ1-tnpA-res-strA-strB-merE-R-*IS*4321R* and IS*26-tnfxB1*-*tmexCD1-toprJ1-tnpA-res-strA-strB-*ΔIS*903B* (Fig. 4). All failed assemblies of plasmids in this study were caused by tandem repeats of *tmexCD1-toprJ1* and shared a core genetic structure that belonged to subtype I with an IS*4321R* element downstream of *tmexCD1-toprJ1* (Fig. 4). The core genetic structure of *tmexCD1-toprJ1* in plasmids pSZP4-9-2-tmexCD and pYTF44-84k-tmexCD was in congruence with that in plasmids pMH15-269M_1, p18-29-MDR, pHN111WT-1, and pHNWH61-1, which was characterized by an ΔIS*903B* element downstream of *tmexCD1-toprJ1*. Additionally, we did not find multiple copies of *tmexCD1-toprJ1* in these plasmids. According to the genetic environment of *tmexCD1-toprJ1* in Klebsiella species plasmids, the mobilization of *tmexCD1-toprJ1* might be driven by both integrases and IS*26*. Given that insertion sequence (IS) elements and integrases play vital roles in the dissemination of antimicrobial resistance genes (3), further diffusion of *tmexCD1-toprJ1* as a result of other integrases and IS elements should be taken into consideration.

### Diversity of tandem repeat regions and copy number heterogeneity of *tmexCD1-toprJ1* in plasmids.

**FIG 5:**
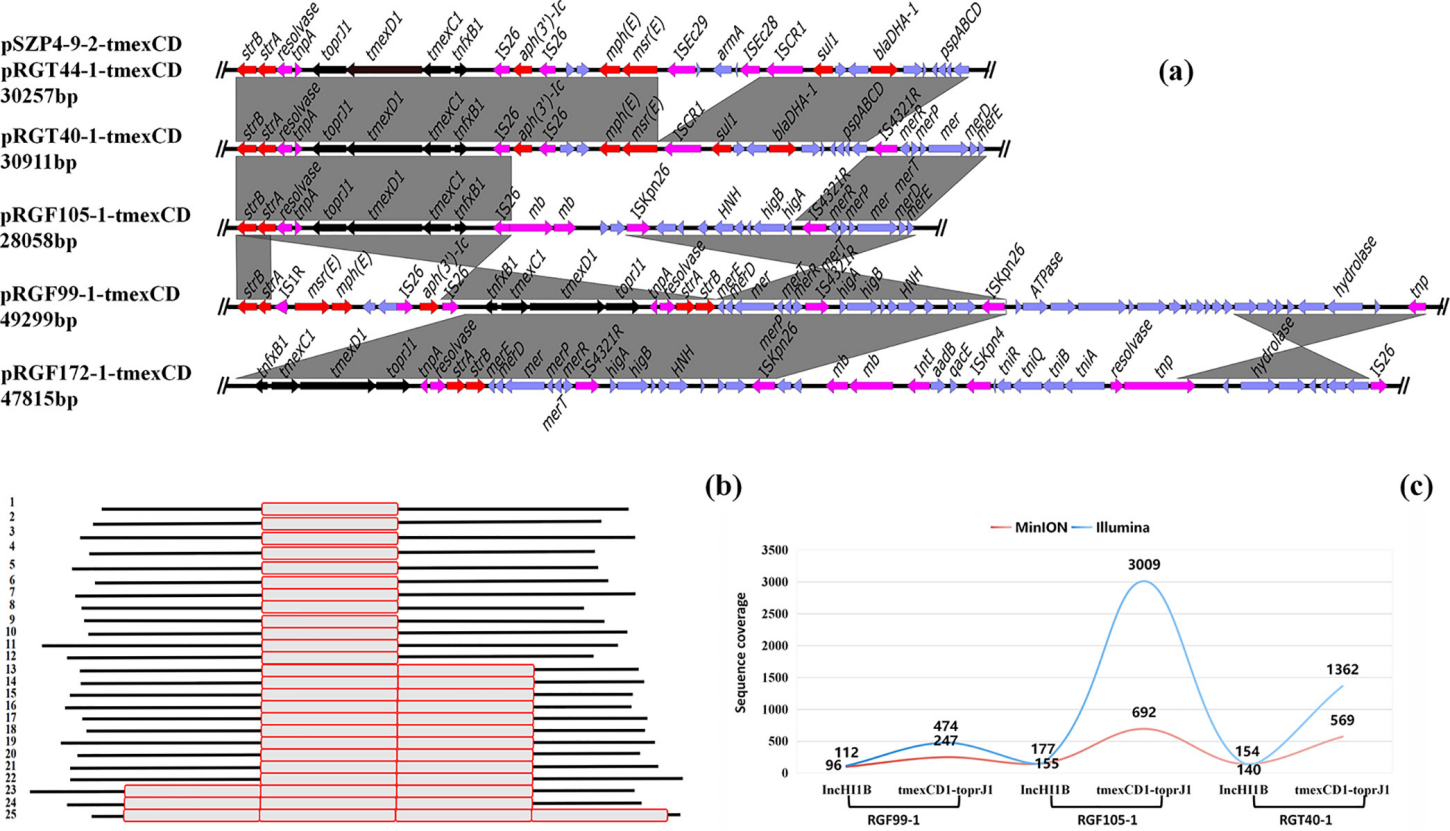
Diversity and heterogeneity of tandem repeat regions of *tmexCD1-toprJ1*. (a) Linear comparison of the genetic structure of *tmexCD1-toprJ1*-bearing repeat regions with that of *tmexCD1-toprJ1* in plasmids pSZP4-9-2-tmexCD and pRGT44-1-tmexCD. (b) Diagram showing copy number heterogeneity of *tmexCD1-toprJ1-*bearing regions, as detected in raw reads spanning the entire tandem repeat arrays of RGT40-1 strains. The red box represents the *tmexCD1-toprJ1*-bearing region. (c) Copy numbers of *tmexCD1-toprJ1* in different strains, as determined by different sequencing strategies.

All unfinished *tmexCD1-toprJ1*-bearing plasmids with circular forms were assembled into two contigs and characterized for plasmid backbones and *tmexCD1-toprJ1*-bearing regions, respectively. To determine the reason for the assembly failure, we analyzed Nanopore raw long-read data. Four kinds of tandem repeat regions containing *tmexCD1-toprJ1* with different sizes were detected in these plasmids (Fig. 5a). However, no reads could span the entire tandem repeat arrays because of the long repeat regions. We found evident differences in the resistance genes and IS elements in these genetic regions when comparing tandem repeat regions with the corresponding regions in pSZP4-9-2-tmexCD and pRGT44-1-tmexCD. Abundant mobile elements in these regions might result in more diverse genetic environments. Furthermore, an IS*4321R* element downstream of *tmexCD1-toprJ1* was absent in pSZP4-9-2-tmexCD and pRGT44-1-tmexCD but existed in these repeat regions. We noticed this genetic feature in the above-described analysis of core genetic structures. Therefore, we speculated that the IS*4321R* element was probably responsible for the formation of tandem repeat regions. A previous study reported that two direct IS*26*-flanked pseudo-compound transposons could excise and form a translocatable unit (TU), which could then be reinserted into the chromosome adjacent to IS*26* and form a tandem array of TUs (36). In this study, we observed a similar structure containing *tmexCD1-toprJ1* flanked by two direct IS*4321R* elements with the genetic arrangement IS*4321R*-genes-*tmexCD1-toprJ1*-genes-IS*4321R* and multiple copies of these regions. The mechanism of tandem repeat unit formation that we detected was likely comparable to that in the previous study but mediated by IS*4321R* rather than IS*26*. Hence, such a TU might integrate into IS*4321R* in other regions, thus expediting the dissemination of *tmexCD1-toprJ1*.

Next, we investigated the distribution of four different tandem repeat regions in all K. pneumoniae strains isolated from the Nantong slaughterhouse (Fig. 1). The prevalence of the 28,058-bp repeat unit was the highest in *tmexCD1-toprJ1*-positive K. pneumoniae, followed by the 30,911-bp repeat unit. The 49,229-bp and 47,815-bp repeat units were found only in RGF99-1 and RGF152-1, respectively (Fig. 1). Various degrees of exogenous gene duplication usually create different growth pressures for bacteria, which might affect the prevalence of tandem repeat units with different sizes. In addition, among the slaughterhouse samples, we found different tandem repeat units present in strains that evolved from one ancestor as well as one kind of tandem repeat unit that appeared in different strains (Fig. 1). This result indicated that the generation of tandem repeat units was dynamic and might be affected by bacterial evolution under different foreign conditions.

Nanopore sequencing can directly detect the input DNA molecule and produce ultralong reads. To investigate the heterogeneity of tandem repeat units, we performed plasmid extractions on three strains harboring different kinds of tandem repeat units and sequenced them using the Nanopore MinION platform. We obtained 477, 829, and 572 MB of plasmid sequencing data from RGT40-1, RGF05-1, and RGF99-1, respectively. We then counted the number of tandem repeat units on individual reads from different strains that spanned the entire tandem repeat array (Table S4). Different tandem repeat units were observed in different reads (Fig. 5b). According to the reads, one tandem repeat array was most common in the three samples. The longest read, spanning four tandem repeat arrays, was detected in pRGF40-1-tmexCD. Subsequently, we counted the mean copy numbers of *tmexCD1-toprJ1* from MinION data and Illumina data using the replicon gene of these plasmids as a reference. The mean copy numbers of *tmexCD1-toprJ1* were lower in MinION data than in Illumina data (Fig. 5c), which is consistent with a similar study (37). Amplification losses likely occurred during enrichment culture with no selection pressure. In order to verify this inference, we counted the copy numbers of *tmexCD1-toprJ1* in pRGT40-1-tmexCD with no selection pressure and with tigecycline selection pressure after a 72-h enrichment culture. A 10-fold gap of copy numbers of *tmexCD1-toprJ1* was observed under the two conditions, demonstrating that the copy numbers of *tmexCD1-toprJ1* can be enriched with tigecycline selection pressure.

10.1128/mSystems.00702-21.8 TABLE S4 Counts of Nanopore long reads spanning entire tandem repeat units. Download Table S4, DOCX file, 0.02 MB. Copyright © 2021 Peng et al. 2021 Peng et al. https://creativecommons.org/licenses/by/4.0/ This content is distributed under the terms of the Creative Commons Attribution 4.0 International license.

### Epidemiological features and genetic structural diversity of *tmexCD1-toprJ1*.

Few studies have examined the epidemiological features and genetic structural diversity of *tmexCD1-toprJ1* since it was first reported. Many complete bacterial genomes and plasmids containing *tmexCD1-toprJ1* were recently deposited in the NCBI nr database. Hence, all *tmexCD1-toprJ1-*bearing sequences consist of chromosomes and plasmids from the NCBI nr database that were retrieved as of 20 January 2021 and subsequently analyzed (Table S3). The majority of *tmexCD1-toprJ1*-positive strains in the NCBI nr database were Pseudomonas spp. and *Aeromonas* spp., with the detection of *tmexCD1-toprJ1* in their plasmids or chromosomes. The *tmexCD1-toprJ1*-positive strains of *Enterobacterales* were exclusively limited to Klebsiella spp. Furthermore, most positive strains were identified from human sources, which suggests that *tmexCD1-toprJ1*-mediated tigecycline resistance is a serious issue in clinical treatment. It is noteworthy that the spread of positive strains was not limited by space and time (Table S3). Efficient approaches to prevent the further dissemination of *tmexCD1-toprJ1* need to be developed urgently.

Next, we investigated the genetic structure of *tmexCD1-toprJ1* in plasmids harbored by Klebsiella spp. We further explored and summarized the genetic structural diversity of *tmexCD1-toprJ1* in other sequences of plasmids and chromosomes. The distinction of the core genetic environment of *tmexCD1-toprJ1* was unapparent from chromosomes and plasmids. However, many regions with a size of ∼15 kb containing *tmexCD1-toprJ1* gene clusters and two *int* genes were observed, which were inserted into the *umuC* gene (Fig. S4). A similar structure was also detected in plasmids of Klebsiella spp. (Fig. 4), which suggested that the mobilization of the *tmexCD1-toprJ1* gene cluster was due to the two *int* genes, while the *umuC* gene was likely an integration hot spot for the two integrases. The *umuCD* gene, also called *rumBC*, was the insertion site of variable region III of SXT/R391 ICEs (5, 38). Variable region III consisted of many exogenous genes, including resistance genes such as *tmexCD1-toprJ1* (31), *tet*(X) (39, 40), and *bla*_NDM-5_ (41). Furthermore, *umuCD* encodes an error-prone DNA polymerase, which is usually found in chromosomes and plasmids of bacteria (42). The ubiquity of *umuCD* seems to have created favorable conditions for the spread of the *tmexCD1-toprJ1* gene cluster. Since abundant mobile genes and resistance genes were observed around *tmexCD1-toprJ1* (Fig. S4), it is reasonable to presume that the *tmexCD1-toprJ1* gene cluster did not originate from Pseudomonas spp. or *Aeromonas* spp. Additional studies should be undertaken to obtain more information about the origin and epidemiological features of the *tmexCD1-toprJ1* gene cluster.

10.1128/mSystems.00702-21.4 FIG S4 Core genetic environment of *tmexCD1-toprJ1* in plasmid or chromosome sequences in the NCBI nr database. The sequence identifiers are GenBank accession numbers. (a) Genetic structure of *tmexCD1-toprJ1* in chromosomes of Pseudomonas spp. (b) Genetic structure of *tmexCD1-toprJ1* in plasmids of Pseudomonas spp. (c) Genetic structure of *tmexCD1-toprJ1* in *Aeromonas* spp., Citrobacter freundii, and an uncultured bacterium. Download FIG S4, TIF file, 0.4 MB. Copyright © 2021 Peng et al. 2021 Peng et al. https://creativecommons.org/licenses/by/4.0/ This content is distributed under the terms of the Creative Commons Attribution 4.0 International license.

### Conclusion.

In summary, our results demonstrate that the prevalence of *tmexCD1-toprJ1* in the pork production chain was evidently different in each part and lower than that previously reported for *tet*(X). The specific host range and limited plasmid vector have so far prevented the wide spread of *tmexCD1-toprJ1*. Additionally, IS elements and integrases were also drivers of the horizontal transmission of *tmexCD1-toprJ1* along with plasmids. Furthermore, a variety of “translocatable units” were detected in a group of similar plasmids, which might facilitate the dissemination of *tmexCD1-toprJ1*. However, the mobilization of *tmexCD1-toprJ1* by transferase or translocatable units was not confirmed by experiments. Although the current prevalence of *tmexCD1-toprJ1* in *Enterobacterales* is not yet critical, there is an urgent necessity to develop and implement a reasonable use of antibiotics such as tetracyclines, together with other effective strategies, to prevent the spread of antibiotic resistance genes.

## MATERIALS AND METHODS

### Sample collection and bacterial isolates.

The samples in this study were collected from swine farms, slaughterhouses, and retail pork from different areas of China from 2018 to 2020. In May 2018, a total of 215 samples, including 55 fecal samples, 54 anal swab samples, 54 nasal swab samples, 24 sewage samples, 19 soil samples, and 9 dust samples, were collected from two swine farms in Nantong, Jiangsu Province. In May 2019, 240 samples, consisting of 182 swine fecal samples, 22 swine carcass samples, 11 ground blood samples, 10 wastewater samples, and 15 soil samples, were collected from a slaughterhouse in Nantong, Jiangsu Province. During the summer of 2019, 139 retail pork samples were purchased from supermarkets, shops, and food markets in different cities in China. In October 2020, 88 samples, including 75 swine fecal samples, 9 wastewater samples, and 4 ground blood samples, were collected from a slaughterhouse in Yangzhou, Jiangsu Province. All of the solid samples were collected using individual sterile feces collectors, and the total weight of each collected sample was ∼2 g. Liquid samples were collected using 5-ml sterile centrifuge tubes. The swab samples were collected using sterile swabs and deposited into 5-ml sterile centrifuge tubes. The collected samples were stored in iceboxes at a low temperature and transported to the laboratory instantly for the following processing steps.

Solid samples (1 g), such as feces, soil, or pork, and liquid samples (1 ml), such as wastewater, blood, or cotton swabs (surface samples), were incubated in 5 ml LB broth supplemented with tigecycline (2 mg/liter) for 6 h to enrich the tigecycline-resistant microbiota. The tigecycline-resistant colonies were screened using MacConkey agar plates supplemented with tigecycline (4 mg/liter). Subsequently, the *tmexCD1-toprJ1*-positive strains were screened from all tigecycline-resistant isolates using a PCR method with previously reported primers (43).

### Antimicrobial susceptibility testing and plasmid transfer experiment.

The MICs of all *tmexCD1-toprJ1*-positive isolates were tested by broth microdilution according to Clinical and Laboratory Standards Institute (CLSI) guidelines (56). E. coli ATCC 25922 was used for quality control. The resistance breakpoints for tigecycline were interpreted as >2 mg/liter according to European Committee on Antimicrobial Susceptibility Testing (EUCAST) (http://www.eucast.org/clinical_breakpoints/) breakpoints due to data deficiency in the CLSI guidelines. In order to corroborate the transferability of the *tmexCD1-toprJ1* gene cluster, we conducted conjugation experiments using E. coli J53 (Azi^r^) and C600 (Rif^r^) and K. pneumoniae HS11286YZ6 (Hm^r^) as recipients. First, the donor and recipient strains were cultured to the logarithmic growth phase with an optical density at 600 nm (OD_600_) of 0.4 in LB broth, mixed at a ratio of 1:1, and cultured overnight in a 15-fold volume of fresh LB broth or on LB agar plates. Afterwards, the transconjugants were screened on LB agar plates containing sodium azide (300 mg/liter), rifampin (300 mg/liter), or hygromycin (300 mg/liter) and tigecycline (2 mg/liter), after which they were confirmed by PCR targeted at the *tmexCD1-toprJ1* gene cluster and 16S rRNA or ST alleles. From those *tmexCD1-toprJ1*-positive plasmids in strains that we were unable to perform conjugation assays on, we selected part of them to conduct electroporation experiments (44). The electroporation conditions were adjusted according to the DNA fragment size (45). Specifically, cuvettes with a 2-mm gap were placed on ice to chill, and electrocompetent cells were thawed on ice; next, 50 μl of electrocompetent cells was added to the cuvettes on ice. Electroporation conditions were 200 Ω, 1.8 kV, and 25 μF. The transconjugants were screened on LB agar plates containing tigecycline (2 mg/liter).

### Genome extraction and sequencing.

The genomes of *tmexCD1-toprJ1*-positive isolates were extracted using the FastPure bacterial DNA isolation minikit (catalog no. DC103; Vazyme) according to the manufacturer’s instructions. DNA quality was evaluated using a Qubit 4 fluorometer and a NanoDrop instrument (Thermo Scientific). The genomic DNA of all *tmexCD1-toprJ1*-positive isolates was subjected to short-read sequencing (2 by 150 bp) using the Illumina HiSeq 2500 platform. In order to investigate the *tmexCD1-toprJ1*-bearing plasmids and genetic context diversity, most strains were sequenced using the Oxford Nanopore Technologies MinION platform. Subsequently, we obtained the complete genome sequences of the isolates sequenced by Illumina and Nanopore by hybrid assembly using Unicycler (46, 47).

### Plasmid extraction, sequencing, and single-molecule analysis.

Most *tmexCD1-toprJ1*-bearing plasmids failed to provide complete sequences, as they were affected by tandem repeats of the *tmexCD1-toprJ1*-containing region. To explore the diversity of repeat regions and the copy number heterogeneity of *tmexCD1-toprJ1* in different plasmids, three representative plasmids with repeat regions of different sizes were selected to perform ultralong Nanopore MinION sequencing. The plasmid DNA was extracted using the Qiagen Plasmid Midi kit (Qiagen, Germany) after culture overnight in LB broth (100 ml). The sequencing library of plasmid DNA was prepared using the RBK004 barcoding library preparation kit according to the manufacturer’s protocol. In order to generate longer reads, the incubating time for plasmid DNA fragmentation was modified from 1 min to 40 s. The library was then sequenced on an R9.4 flow cell on the MinION Mk1c device running MinKnow. The fast5 files generated by MinKnow were base called using Guppy in the high-accuracy mode. The *tmexCD1-toprJ1*-bearing reads were extracted using seqkit tools. The copy numbers of *tmexCD1-toprJ1* in single-molecule reads were counted by BLAST analysis using CLC genomics workbench v10.0.1. The copy number heterogeneity of *tmexCD1-toprJ1* was assessed by the reads that spanned the entire tandem repeat arrays.

### Bioinformatics analysis.

Antimicrobial resistance genes, IS elements, and plasmid replicon types were identified by CGE Services (https://cge.cbs.dtu.dk/services/). The draft genomes were annotated by Prokka (48). Functional annotation of the complete genome sequences was achieved using RAST (http://rast.nmpdr.org/) automatically and then modified manually. MLST of assembled strain genomes was performed using the mlst tool (https://github.com/tseemann/mlst). The phylogenetic tree of strains was constructed using Roary (49) and FastTree (50), based on SNPs of core genomes, and embellished using the online tool iTOL v4 (51). The SNPs in different strains were analyzed using the snp-dists tool (https://github.com/tseemann/snp-dists). The BRIG and Easyfig tools were used to visualize the plasmid comparisons (52) and genetic context comparisons (53). A heat map was constructed using TBtools (54). The chromosomes and plasmids carrying *tmexCD1-toprJ1* in the NCBI nr database were found by BLAST analysis and downloaded by the Web browser via HTTP. The copy number of *tmexCD1-toprJ1* was defined as the ratio of *tmexCD1-toprJ1* sequencing coverage to plasmid replicon gene sequencing coverage. For strains sequenced by the Illumina short-read platform, we counted the coverage of contigs harboring *tmexCD1-toprJ1* or the plasmid replicon gene directly from draft genome assembly statistics. The coverage of contigs was the K-mer coverage for the highest K value used in short-read assemblies by SPAdes. For strains sequenced by the Nanopore long-read platform, we mapped the Nanopore long-read raw data against *tmexCD1-toprJ1* using minimap2 (55) to calculate the coverage of *tmexCD1-toprJ1*. Meanwhile, the coverage of the plasmid replicon gene was calculated in the same way.

### Data availability.

The genome sequences in this study were deposited into the National Center for Biotechnology Information database under BioProject accession no. PRJNA719697.
