# Supplementary figures and images for: Plasmids Shape the Current Prevalence of tmexCD1-toprJ1 among Klebsiella pneumoniae in Food Production Chains
Source: mSystems. 2021 Oct 5;6(5):e00702-21. doi: 10.1128/mSystems.00702-21 (PMC8547460; doi:10.1128/mSystems.00702-21)

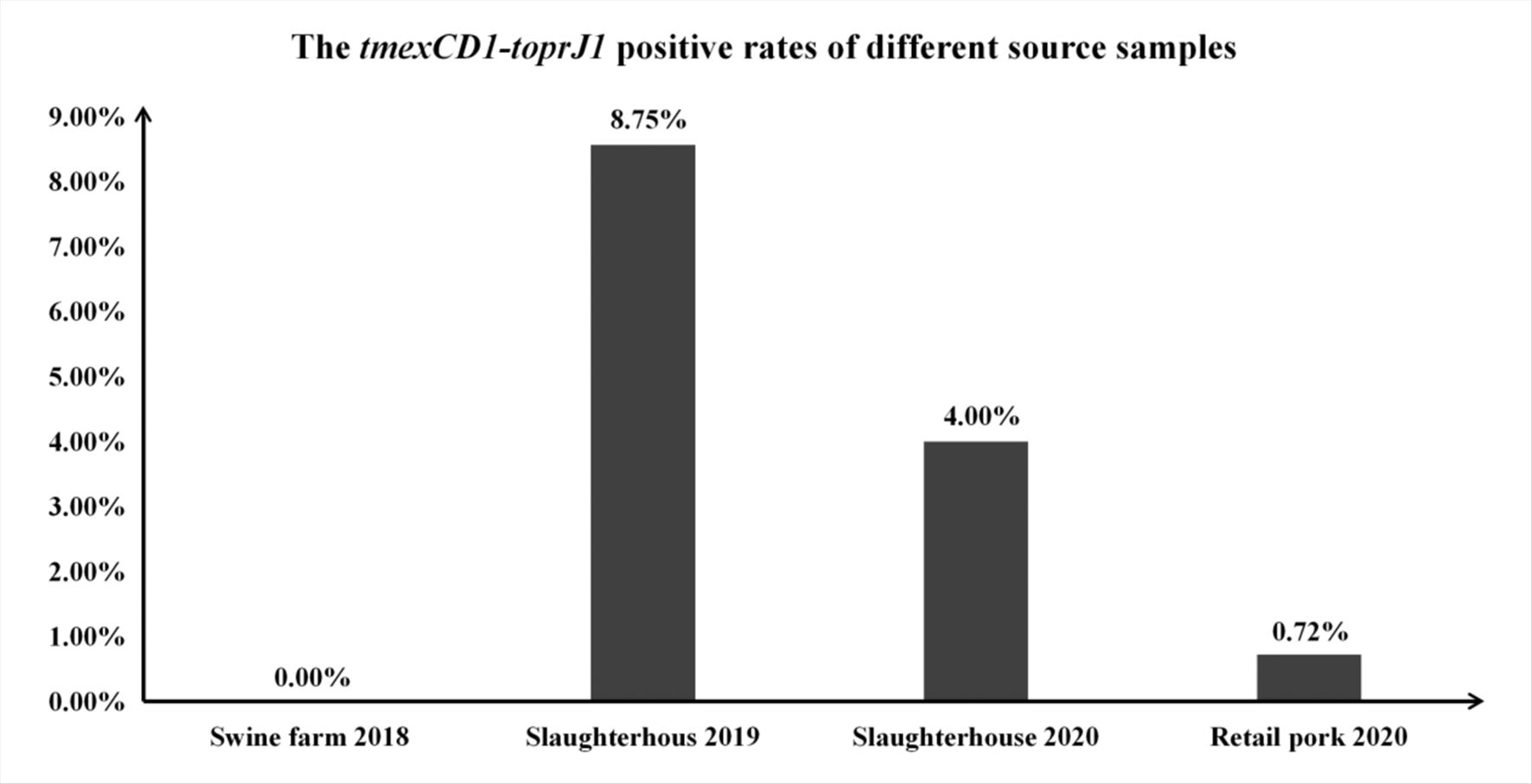

Supplement: FIG S1 [file msystems.00702-21-sf001.tif]

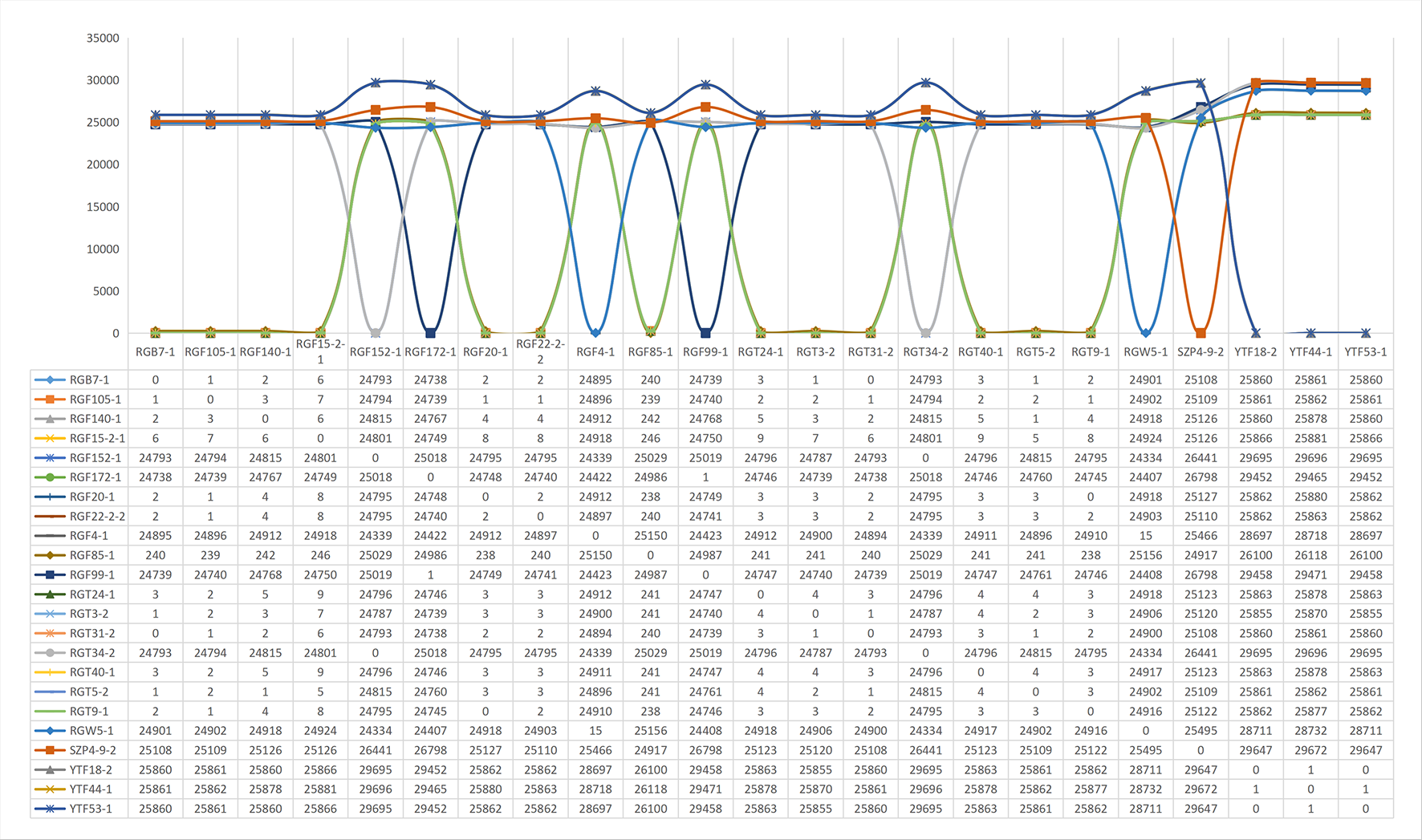

Supplement: FIG S2 [file msystems.00702-21-sf002.tif]

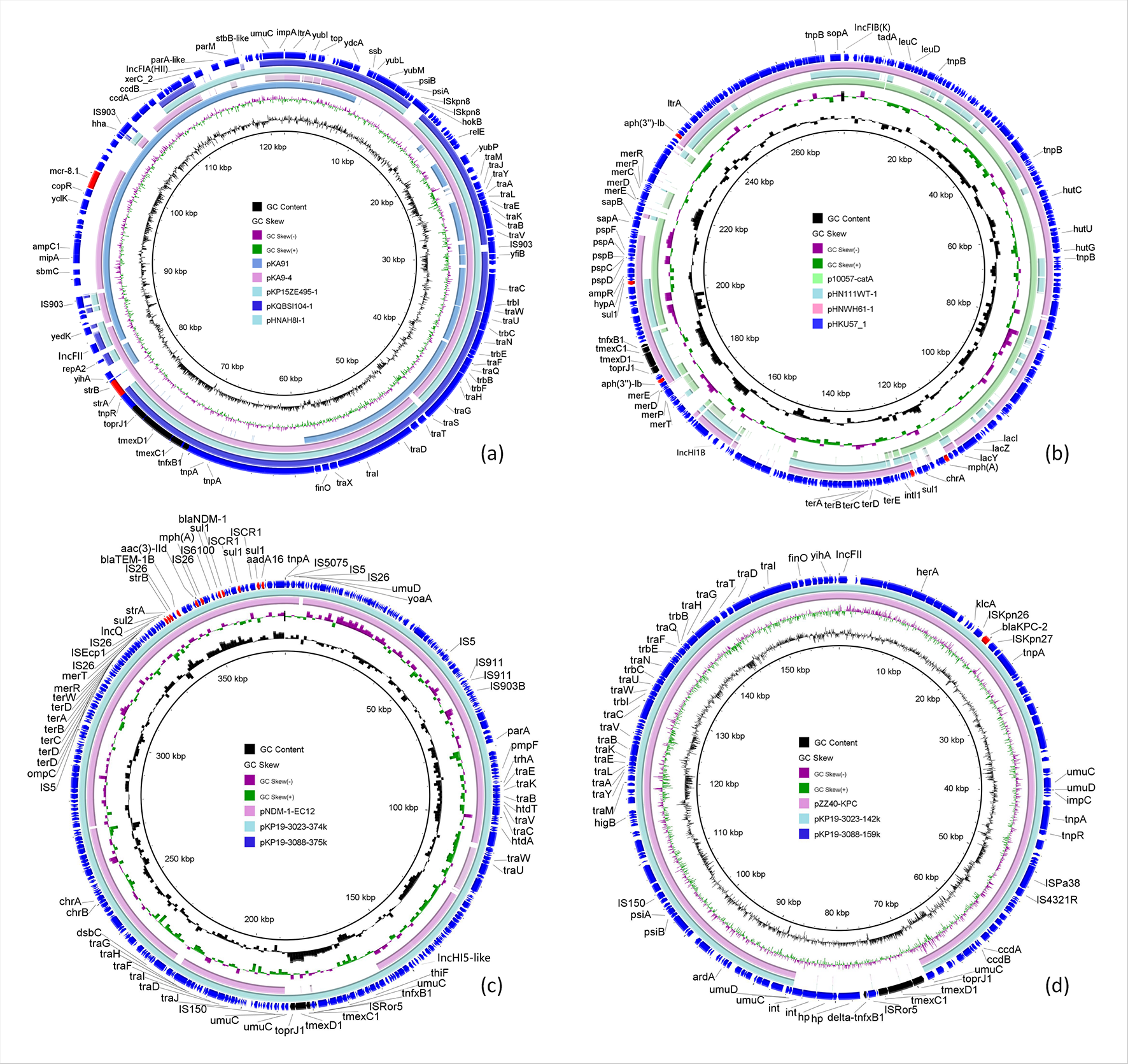

Supplement: FIG S3 [file msystems.00702-21-sf003.tif]

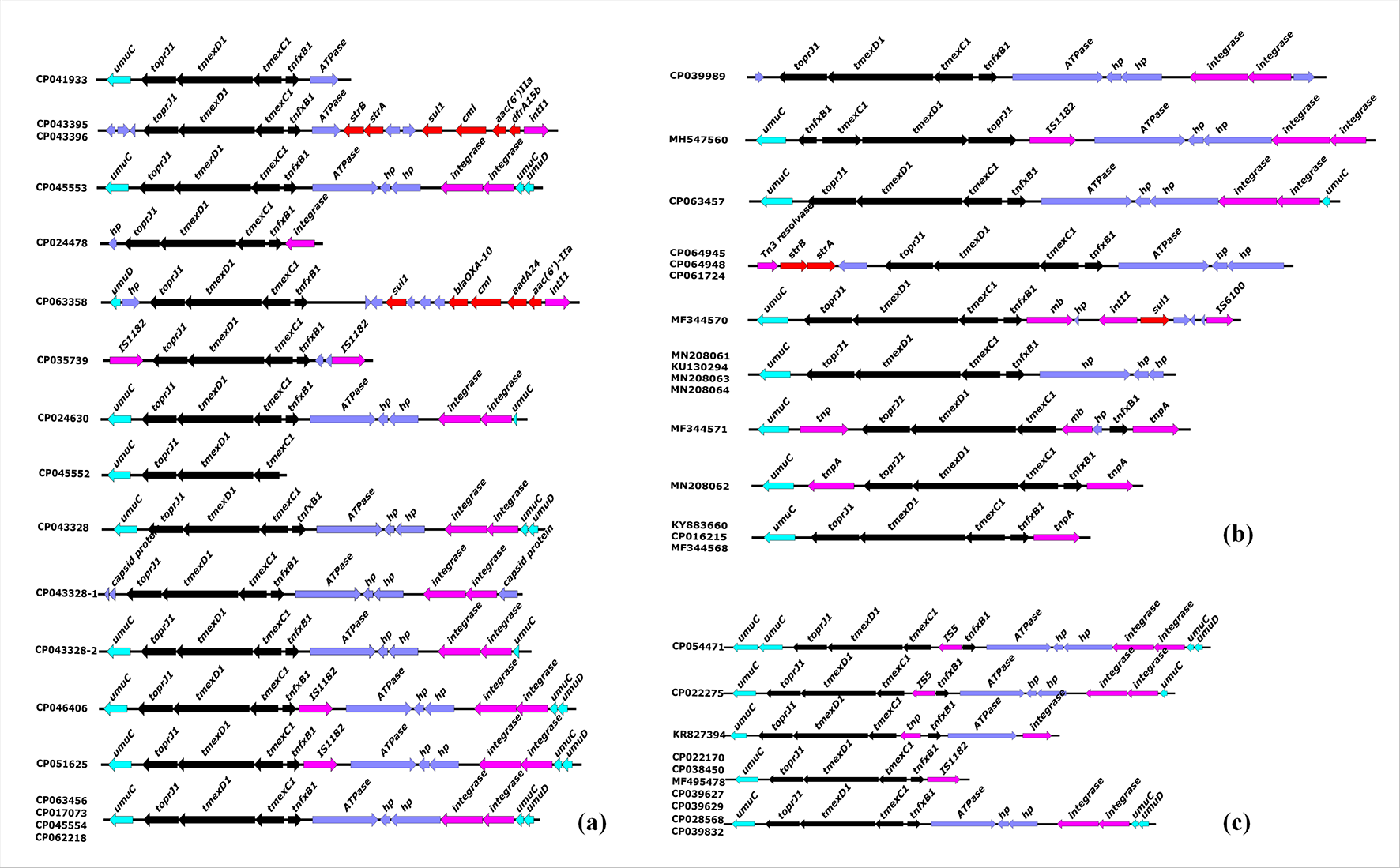

Supplement: FIG S4 [file msystems.00702-21-sf004.tif]
