# Supplementary material for: Plasmids Shape the Current Prevalence of tmexCD1-toprJ1 among Klebsiella pneumoniae in Food Production Chains
Source: mSystems. 2021 Oct 5;6(5):e00702-21. doi: 10.1128/mSystems.00702-21 (PMC8547460; doi:10.1128/mSystems.00702-21)
Supplement: TABLE S1 [file msystems.00702-21-st001.docx]

| **The samples collected from two pig farms in Nantong City in May 2018** | | | | |
| --- | --- | --- | --- | --- |
| Sources | Number of samples | Number of *tmexCD1-toprJ1* positive strains | Number of positive samples | Positive rates^a^ |
| Feces | 55 | 0 | 0 | 0.00% |
| Anal swabs | 54 | 0 | 0 | 0.00% |
| Nose swabs | 54 | 0 | 0 | 0.00% |
| Sewage | 24 | 0 | 0 | 0.00% |
| Soil | 19 | 0 | 0 | 0.00% |
| Dust | 9 | 0 | 0 | 0.00% |
| Total | 215 | 0 | 0 | 0.00% |
| **Samples from a slaughterhouse in Nantong City in May 2019** | | | | |
| Sources | Number of samples | Number of *tmexCD1-toprJ1* positive strains | Number of positive samples | Positive rates^a^ |
| Soil | 15 | 0 | 0 | 0.00% |
| Blood | 11 | 1 | 1 | 9.09% |
| Wastewater | 10 | 1 | 1 | 10.00% |
| Carcass | 22 | 7 | 7 | 31.82% |
| Feces | 182 | 12 | 12 | 6.59% |
| Total | 240 | 21 | 21 | 8.75% |
| **Samples from retail pork collected in different cities of China in 2019** | | | | |
| Sources | Number of samples | Number of *tmexCD1-toprJ1* positive strains | Number of positive samples | Positive rates^a^ |
| Shandong | 13 | 0 | 0 | 0.00% |
| Shanxi | 13 | 0 | 0 | 0.00% |
| Sichuan | 15 | 0 | 0 | 0.00% |
| Guangdong | 12 | 1 | 1 | 8.33% |
| Gansu | 12 | 0 | 0 | 0.00% |
| Henan | 18 | 0 | 0 | 0.00% |
| Shanghai | 13 | 0 | 0 | 0.00% |
| Anhui | 12 | 0 | 0 | 0.00% |
| Hebei | 19 | 0 | 0 | 0.00% |
| Zhejiang | 12 | 0 | 0 | 0.00% |
| Total | 139 | 1 | 1 | 0.72% |
| **The samples collected from a slaughterhouse in Yangzhou City in Oct 2020** | | | | |
| Sources | Number of samples | Number of *tmexCD1-toprJ1* positive strains | Number of positive samples | Positive rates^a^ |
| Blood | 4 | 0 | 0 | 0.00% |
| Wastewater | 9 | 0 | 0 | 0.00% |
| Feces | 75 | 3 | 3 | 4.00% |
| Total | 88 | 3 | 3 | 4.00% |

^a^ Positive rate= Number of positive samples/Number of samples.
