# Supplementary material for: Plasmids Shape the Current Prevalence of tmexCD1-toprJ1 among Klebsiella pneumoniae in Food Production Chains
Source: mSystems. 2021 Oct 5;6(5):e00702-21. doi: 10.1128/mSystems.00702-21 (PMC8547460; doi:10.1128/mSystems.00702-21)
Supplement: TABLE S2 [file msystems.00702-21-st002.docx]

| IDs | Sources | Species |  | Antimicrobials | | | | | | | | | | | | | |
| --- | --- | --- | --- | --- | --- | --- | --- | --- | --- | --- | --- | --- | --- | --- | --- | --- | --- |
|  |  |  | TIG | TIG+NMP | MIN | OXY | DOX | TET | MEM | CL | CFF | AMX | KAN | STR | FFC | ENR | CIP |
| RGT34-2 | Carcass | *K. pneumoniae* | 32 | 2 | 64 | >256 | >256 | >256 | ≤0.25 | 0.5 | 4 | >128 | >256 | >128 | >128 | 4 | 2 |
| RGF172-1 | Faeces | *K. pneumoniae* | 32 | 4 | 256 | >256 | >256 | >256 | ≤0.25 | ≤0.25 | >128 | >128 | >256 | >128 | >128 | 16 | 32 |
| RGT31-2 | Carcass | *K. pneumoniae* | 32 | 2 | 128 | >256 | 128 | >256 | ≤0.25 | 0.5 | 32 | >128 | >256 | >128 | 16 | 8 | 32 |
| RGF105-1 | Faeces | *K. pneumoniae* | 32 | 4 | 256 | >256 | 64 | >256 | ≤0.25 | ≤0.25 | 8 | >128 | >256 | >128 | 8 | 8 | 16 |
| RGT24-1 | Carcass | *K. pneumoniae* | 32 | 2 | >256 | >256 | >256 | >256 | ≤0.25 | ≤0.25 | 2 | >128 | >256 | >128 | 8 | 4 | 8 |
| RGF20-1-1 | Faeces | *K. pneumoniae* | 32 | 2 | 128 | >256 | 128 | >256 | ≤0.25 | 0.5 | 4 | >128 | >256 | >128 | 16 | 8 | 16 |
| RGF152-1 | Faeces | *K. pneumoniae* | 32 | 2 | 64 | >256 | 64 | >256 | ≤0.25 | ≤0.25 | 4 | >128 | >256 | >128 | >128 | 4 | 2 |
| RGF15-2-1 | Faeces | *K. pneumoniae* | 32 | 2 | 128 | >256 | 128 | >256 | ≤0.25 | ≤0.25 | 32 | >128 | >256 | >128 | 16 | 8 | 8 |
| RGT9-1 | Carcass | *K. pneumoniae* | 32 | 2 | 128 | >256 | 64 | >256 | ≤0.25 | ≤0.25 | 16 | >128 | >256 | >128 | 2 | 8 | 8 |
| RGT3-2 | Carcass | *K. pneumoniae* | 32 | 2 | 128 | >256 | 64 | >256 | ≤0.25 | ≤0.25 | 16 | >128 | >256 | >128 | 32 | 16 | 8 |
| RGT22-2-2 | Carcass | *K. pneumoniae* | 32 | 2 | >256 | >256 | 64 | >256 | ≤0.25 | ≤0.25 | 32 | >128 | >256 | >128 | 16 | 16 | 16 |
| RGF11-2 | Faeces | *P. mirabilis* | 32 | 2 | >256 | >256 | >256 | >256 | ≤0.25 | >128 | >128 | >128 | >256 | >128 | >128 | 32 | 64 |
| RGF134-1 | Faeces | *P. mirabilis* | 32 | 2 | 128 | >256 | 128 | 128 | ≤0.25 | >128 | >128 | >128 | >256 | >128 | >128 | >128 | 64 |
| RGW5-1 | Wastewater | *K. pneumoniae* | 16 | 2 | 64 | >256 | 128 | >256 | ≤0.25 | ≤0.25 | >128 | >128 | >256 | >128 | >128 | 8 | 16 |
| RGF4-1 | Faeces | *K. pneumoniae* | 32 | 2 | 64 | >256 | 64 | >256 | ≤0.25 | ≤0.25 | >128 | >128 | >256 | >128 | >128 | 2 | 16 |
| RGB7-1 | Blood | *K. pneumoniae* | 32 | 2 | 128 | >256 | 64 | >256 | ≤0.25 | ≤0.25 | 32 | >128 | >256 | >128 | 8 | 8 | 16 |
| RGT40-1 | Carcass | *K. pneumoniae* | 32 | 2 | 128 | >256 | 64 | >256 | ≤0.25 | ≤0.25 | 32 | >128 | >256 | >128 | 8 | 16 | 8 |
| RGF140-1 | Faeces | *K. pneumoniae* | 32 | 2 | 128 | >128 | 128 | >128 | ≤0.25 | ≤0.25 | >128 | >128 | >256 | >128 | 32 | 8 | 8 |
| RGT5-2 | Carcass | *K. pneumoniae* | 32 | 2 | 64 | >128 | 64 | >128 | ≤0.25 | ≤0.25 | 1 | >128 | >256 | >128 | 16 | 4 | 4 |
| RGF85-1 | Faeces | *K. pneumoniae* | 16 | 2 | 64 | >128 | 64 | >128 | ≤0.25 | ≤0.25 | 2 | >128 | >256 | >128 | 8 | 4 | 8 |
| RGF99-1 | Faeces | *K. pneumoniae* | 32 | 2 | 32 | >128 | 64 | >128 | ≤0.25 | ≤0.25 | >128 | >128 | >256 | >128 | >128 | 2 | 4 |
| YTF44-1 | Faeces | *K. pneumoniae* | 32 | 2 | >256 | >128 | 64 | >128 | ≤0.25 | ≤0.25 | 8 | >128 | >256 | ≤0.25 | >128 | >128 | ≤0.25 |
| YTF53-1 | Faeces | *K. pneumoniae* | 32 | 2 | >256 | >128 | 128 | >128 | ≤0.25 | ≤0.25 | 32 | >128 | >256 | >128 | >128 | >128 | >128 |
| YTF18-2 | Faeces | *K. pneumoniae* | 32 | 2 | >256 | >128 | 128 | >128 | ≤0.25 | ≤0.25 | 4 | >128 | >256 | >128 | >128 | >128 | >128 |
| SZP4-9-2 | Pork | *K. pneumoniae* | 32 | 2 | >256 | >256 | >256 | >256 | 0.5 | ≤0.25 | 64 | >128 | 64 | >128 | >128 | 64 | 64 |
| TF44-1-DH5α | / | 1. *coli* | 8 | / | 128 | >128 | 64 | >128 | ≤0.25 | ≤0.25 | 4 | >128 | >128 | >128 | 64 | 2 | 4 |

**Abbreviations**: TIG, tigecycline; NMP, 1-(1-naphthylmethyl)-piperazine; MIN, minocyline; OXY, oxytetracycline; DOX, doxycycline; TET, tetracycline; MEM, meropenem; CL, colistin; CFF, ceftiofur; AMX, amoxicillin; KAN, kanamycin; STR, streptomycin; FFC, florfenicol; ENR, enrofloxacin; CIP, ciprofloxacin.
