# Supplementary material for: Plasmids Shape the Current Prevalence of tmexCD1-toprJ1 among Klebsiella pneumoniae in Food Production Chains
Source: mSystems. 2021 Oct 5;6(5):e00702-21. doi: 10.1128/mSystems.00702-21 (PMC8547460; doi:10.1128/mSystems.00702-21)
Supplement: TABLE S3 [file msystems.00702-21-st003.docx]

| Species | Chromosomes or plasmids | Size | Accession. no | Source | Year | Location | Identify | Integration site |
| --- | --- | --- | --- | --- | --- | --- | --- | --- |
| *Klebsiella pneumoniae* | pHNAH8I-1 | 121961 | MK347425 | Chicken | 2017 | China | 100 | *umuC* |
| *Klebsiella quasipneumoniae* | pHN111WT-1 | 220731 | MT647839 | Sewage | / | China | 99.98 | / |
| *Klebsiella quasipneumoniae* | pKQBSI104-1 | 121966 | MT813036 | Homo sapiens | 2017 | China | 99.98 | *umuC* |
| *Klebsiella pneumoniae* | pKP15ZE495-1 | 102569 | MT813035 | Homo sapiens | 2015 | China | 99.98 | *umuC* |
| *Klebsiella pneumoniae* | pHKU57_1 | 273789 | CP063216 | Homo sapiens | 2017 | Hong Kong | 99.98 | *umuC* |
| *Klebsiella pneumoniae* | pMH15-269M_1 | 288040 | AP023338 | Homo sapiens | 2015 | Viet Nam | 99.98 | *umuC* |
| *Klebsiella pneumoniae* | pHNWH61-1 | 256606 | MN099026 | Homo sapiens | / | China | 99.98 | *umuC* |
| *Klebsiella pneumoniae* | pKA9-4 | 120427 | MN832595 | Chicken | / | China | 99.98 | *umuC* |
| *Klebsiella pneumoniae* | p18-29-MDR | 278636 | MK262712 | Urine | 2018 | China | 99.97 | *umuC* |
| *Aeromonas hydrophila* | WCHAH045096 | 5022876 | CP028568 | Sewage | 2015 | China | 97.97 | *umuC* |
| *Pseudomonas* sp. | 14181154 | 5612807 | CP045554 | Catheter | 2014 | China | 97.75 | *umuC* |
| *Pseudomonas aeruginosa* | pBT2436 | 422811 | CP039989 | Adult male sputum | 2013 | Thailand | 97.75 | *umuC* |
| *Pseudomonas aeruginosa* | CCUG 51971 | 7012798 | CP043328 | Urine sample | 2001 | Sweden | 97.75 | *umuC* |
| *Pseudomonas putida* | PP112420 | 6031212 | CP017073 | Homo sapiens | 2011 | China | 97.75 | / |
| *Pseudomonas aeruginosa* | SE5458 | 7157752 | CP046406 | Homo sapiens | 2011 | China | 97.75 | *umuC* |
| *Pseudomonas fulva* | pVIM-24-ZDHY414 | 589460 | CP064948 | Homo sapiens | 2019 | China | 97.75 | *umuC* |
| *Pseudomonas fulva* | pVIM-24-ZDHY316 | 585396 | CP064945 | Homo sapiens | 2019 | China | 97.75 | *umuC* |
| *Pseudomonas putida* | SWMUB4 plasmid | 199972 | CP063457 | Homo sapiens | 2018 | China | 97.75 | *umuC* |
| *Pseudomonas putida* | SWMUB4 | 5824126 | CP063456 | Homo sapiens | 2018 | China | 97.75 | *umuC* |
| *Pseudomonas putida* | SY153 | 5604657 | CP062218 | Urine | 2012 | China | 97.75 | *umuC* |
| *Pseudomonas* sp. | gcc21 | 3983233 | CP051625 | Deep sea | 2018 | China | 97.73 | *umuC* |
| *Pseudomonas aeruginosa* | pA681-IMP | 397519 | MF344570 | Homo sapiens | / | China | 97.73 | *umuC* |
| *Pseudomonas putida* | pSY153-MDR | 468170 | KY883660 | Homo sapiens | 2012 | China | 97.73 | *umuC* |
| *Pseudomonas aeruginosa* | pBM413 | 423017 | CP016215 | Sputum | 2012 | China | 97.73 | *umuC* |
| *Pseudomonas putida* | pZXPA-20-602k | 602772 | CP061724 | Migratory birds | 2019 | China | 97.73 | *umuC* |
| *Pseudomonas aeruginosa* | p243931-IMP | 392046 | MN208062 | Homo sapiens | 2016 | China | 97.73 | *umuC* |
| *Aeromonas media* | pAeme6 | 199818 | CP038450 | Sludge from bioreactor | 2016 | China | 97.72 | *umuC* |
| *Pseudomonas aeruginosa* | pMKPA34-1 | 95404 | MH547560 | Microbial keratitis | 1997 | India | 97.72 | *umuC* |
| *Aeromonas salmonicida* | pS121-1b | 188161 | MF495478 | / | / | / | 97.72 | *umuC* |
| *Aeromonas salmonicida* | pS121-1a | 195805 | CP022170 | The RAS Atlantic Salmon facility | 2015 | China | 97.72 | *umuC* |
| *Aeromonas caviae* | pAeca1-a | 228716 | CP039629 | Sludge from bioreactor | 2016 | China | 97.72 | *umuC* |
| *Aeromonas caviae* | pAeca1-b | 221458 | CP039627 | Sludge from bioreactor | 2016 | China | 97.72 | *umuC* |
| *Aeromonas caviae* | WCW1-2 | 4684096 | CP039832 | Sewage | 2018 | China | 97.70 | / |
| *Pseudomonas aeruginosa* | p519119-DIM | 407906 | MN208061 | Homo sapiens | 2017 | China | 97.53 | *umuC* |
| *Pseudomonas putida* | p12969-DIM | 409102 | KU130294 | Homo sapiens | / | China | 97.52 | *umuC* |
| *Pseudomonas* sp. | pBJP69-DIM | 407628 | MN208064 | Homo sapiens | 2015 | China | 97.52 | *umuC* |
| *Pseudomonas aeruginosa* | p60503-DIM | 407628 | MN208063 | Homo sapiens | 2016 | China | 97.50 | *umuC* |
| *Pseudomonas* sp. | HLS-6 | 5276694 | CP024478 | Water | 2017 | China | 97.41 | / |
| *Pseudomonas aeruginosa* | p727-IMP | 430173 | MF344568 | Homo sapiens | / | China | 97.16 | *umuC* |
| *Pseudomonas* sp. | BJP69 | 5597068 | CP041933 | Sputum specimen | 2015 | China | 96.90 | *umuC* |
| *Pseudomonas monteilii* | 170620603RE | 5801682 | CP043396 | Drainage | 2017 | China | 96.89 | / |
| *Pseudomonas monteilii* | 170918607 | 5802791 | CP043395 | Homo sapiens | 2017 | China | 96.89 | / |
| *Pseudomonas aeruginosa* | PA59 | 6926363 | CP024630 | Mink | 2010 | China | 96.95 | *umuC* |
| *Pseudomonas stutzeri* | ZDHY95 | 4500524 | CP063358 | Cerebrospinal fluid | 2019 | China | 96.83 | / |
| *Pseudomonas* sp. | 13159349 | 5977292 | CP045553 | Abdominal fluid human | 2013 | China | 96.81 | *umuC* |
| *Pseudomonas aeruginosa* | 1334/14 | 6902135 | CP035739 | Homo sapiens eye | 2014 | Poland | 96.81 | / |
| *Citrobacter freundii* | pBKPC18-1 | 144825 | CP022275 | Sediment around river | 2015 | China | 97.83 | *umuC* |
| *Klebsiella pneumoniae* | pKP19-3023-374k | 374513 | CP063748 | Sputum | 2019 | China | 97.83 | *umuC* |
| *Klebsiella pneumoniae* | pKP19-3088-375k | 375474 | CP063149 | Blood culture | 2019 | China | 97.83 | *umuC* |
| *Klebsiella pneumoniae* | pKP19-3088-159k | 159093 | CP063148 | Blood culture | 2019 | China | 97.83 | *umuC* |
| uncultured bacterium | pKAZ5 | 222486 | KR827394 | Kazipally lake sediment | 2012 | India | 97.74 | *umuC* |
| *Pseudomonas aeruginosa* | pR31014-IMP | 374000 | MF344571 | Homo sapiens | / | China | 97.47 | *umuC* |
| *Pseudomonas aeruginosa* | YT12746 | 6870662 | CP045552 | Homo sapiens | 2012 | China | 97.80 | *umuC* |
