# Supplementary material for: Plasmids Shape the Current Prevalence of tmexCD1-toprJ1 among Klebsiella pneumoniae in Food Production Chains
Source: mSystems. 2021 Oct 5;6(5):e00702-21. doi: 10.1128/mSystems.00702-21 (PMC8547460; doi:10.1128/mSystems.00702-21)
Supplement: TABLE S4 [file msystems.00702-21-st004.docx]

|  | | The read counts of spanned entire tandem repeat units | | | | |
| --- | --- | --- | --- | --- | --- | --- |
| Strain | The size of tandem repeats | One copy | Two copies | Three copies | Four copies | Total |
| RGT40-1 | 28058 bp | 12 | 10 | 2 | 1 | 25 |
| RGF105-1 | 30911 bp | 19 | 8 | 5 | 0 | 32 |
| RGF99-1 | 49229 bp | 0 | 2 | 1 | 0 | 3 |
